# Microarray based genetic profiling of *Staphylococcus aureus* isolated from abattoir byproducts of pork origin

**DOI:** 10.1371/journal.pone.0222036

**Published:** 2019-09-06

**Authors:** Marina Morach, Nadine Käppeli, Mirjam Hochreutener, Sophia Johler, Jérôme Julmi, Roger Stephan, Danai Etter

**Affiliations:** 1 Institute for Food Safety and Hygiene, Vetsuisse Faculty, University of Zurich, Zurich, Switzerland; 2 Institute of Food, Nutrition and Health, ETH Zurich, Zurich, Switzerland; Instituto de Technologia Quimica e Biologica, PORTUGAL

## Abstract

Many parts of pork meat processing are currently not used for human consumption in Switzerland, although they are of great nutritional value. Therefore, data on the occurrence of pathogenic organisms on byproducts is extremely scarce and the prevalence and population structure of *Staphylococcus aureus* on meat processing sidestreams is unknown. Hence, abattoir byproducts of pork origin including ear, forefoot, heart, intestine, liver, rib bone, sternum, bladder, stomach, hind foot and tongue originating from six abattoirs were screened for *S*. *aureus*. The obtained isolates were investigated by *spa* typing and DNA microarray analysis to reveal their genomic profile and population structure. The prevalence of *S*. *aureus* was generally low with a mean of 8%. In total, 40 *S*. *aureus* strains were detected and assigned to 12 *spa* types (t015, t1491, t1778, t091, t337, t899, t2922, t7439, t1333, t208, t4049, t034) and seven clonal complexes (CC1, CC7, CC9, CC30, CC45, CC49, CC398). Detected enterotoxin genes included *sea*, *seb*, *sec*, *seh*, *sel* and *egc* encoded toxin genes *seg*, *sei*, *sem*, *sen*, *seo*, and *seu*. None of the isolates harbored genes conferring methicillin resistance, but *blaZ/I/R* genes causing penicillin resistance were frequently found. In addition, strains from CC398 exhibited *tetM* and *tetK*, conferring tetracycline resistance. Similarity calculations based on microarray profiles revealed no association of clonal complexes with particular body parts, but revealed a certain correspondence of clonal complex and originating abattoir.

## Introduction

Roughly 23 million tons of pork meat are processed in the European Union annually with a rising tendency [1]. A significant proportion of this meat is wasted during processing [2] either due to shortcomings in the handling of sidestreams or due to low consumer acceptance and therefore limited marketability of products. In other parts of the world, especially various Asian regions (e.g. Philippines, China, Korea), pig ear or pig tongue and other byproducts are considered a delicacy of great value (e.g. dishes like Panlasang Pinoy and Jokbal). Also, in Europe, the movement of “nose to tail” eating [3] has gained recognition in gastronomy and among the general public in recent years [4]. It aims at utilizing all parts of an animal, giving special attention to the culinary potential of offal. Currently, information on the safety of such products is limited [5,6], and information on the occurrence of *Staphylococcus aureus* is missing. *S*. *aureus* is a common skin colonizing organism responsible for staphylococcal food poisoning (SFP). In 2015, EFSA reported 434 food-borne outbreaks due to staphylococcal enterotoxins (SE). Of these, 85 outbreaks were associated with meat or meat products [7]. Generally, pork meat production has raised concern due to the transmission of livestock associated- methicillin-resistant *S*. *aureus* (LA-MRSA) from animals to humans [8,9]. The most prevalent MRSA lineage in Europe is CC398, while in Asia CC9 is more frequent [9]. The genetic profiles of *S*. *aureus* isolated from neck, belly, back, and ham of pig carcasses in Switzerland have been reported [10], but little is known about the occurrence of *S*. *aureus* on slaughtering byproducts.

In this study, ear, forefoot, heart, intestine, liver, rib bone, sternum, bladder, stomach, hind foot and tongue of porcine origin were screened for *S*. *aureus* and the detected isolates were further characterized. In order to unravel the genomic population structure of *S*. *aureus* isolates, *spa* typing and DNA microarray analysis were used. The objectives of this study were to determine the prevalence of *S*. *aureus* found on abattoir byproducts of pork origin and to characterize their virulence gene and antibiotic susceptibility profiles.

## Materials and methods

### Sampling, bacterial isolation and DNA extraction

Overall, 524 samples of abattoir byproducts of pork origin such as ear (n = 42), forefoot (n = 56), hind foot (n = 56), heart (n = 42), intestine (n = 20), liver (n = 42), rib bone (n = 56), sternum (n = 56), bladder (n = 56), stomach (n = 56) and tongue (n = 42) from six abattoirs (56–112 samples from each) were screened for *S*. *aureus*. An aliquot of 10 g of the respective sample was homogenized (Stomacher 80 Biomaster, Seward, West Sussex, United Kingdom) with 90 g NaCl (0.9%) solution (Oxoid, Pratteln, Switzerland). An aliquot of 100 μl each was plated on rabbit plasma fibrinogen agar (Oxoid) and incubated for 48 h at 37°C. The resulting detection limit was 100 CFU/g. For cell lysis, lysostaphin from the Staphytype genotyping kit 2.0 (Alere Technologies GmbH, Jena, Germany) was used before DNA was extracted with the Qiagen DNeasy Blood and Tissue Kit (Hilden, Germany) as described in the manufacturer’s instructions.

### DNA microarray analysis

DNA microarray was performed using Staphytype genotyping kit 2.0 (Alere) following the manufacturer's instructions. An ArrayMate reader (Alere) was used for signal acquisition. In addition, the similarity of the virulence and resistance gene profiles was visualized using SplitsTree4 (http://www.splitstree.org/ [11]) as previously described [12].

### *spa* typing

The sequence of the polymorphic X region of the *spa* gene of each *S*. *aureus* isolate was determined as described previously [13] with minor modifications. In short, the *spa* gene was amplified using the GoTaq PCR system (Promega AG, Dübendorf, Switzerland) at the following reaction conditions: i) 5 min at 94°C; ii) 29x [45 s at 94°C; 45 s at 60°C; 90 s at 72°C]; iii) 10 min at 72°C. Sequencing was outsourced to Microsynth (Balgach, Switzerland) and sequences were assigned to *spa* types using the spa-server (http://www.spaserver.ridom.de/; [14]) as previously described by [12].

## Results and discussion

### *S*. *aureus* prevalence in abattoir byproducts

**Fig 1:**
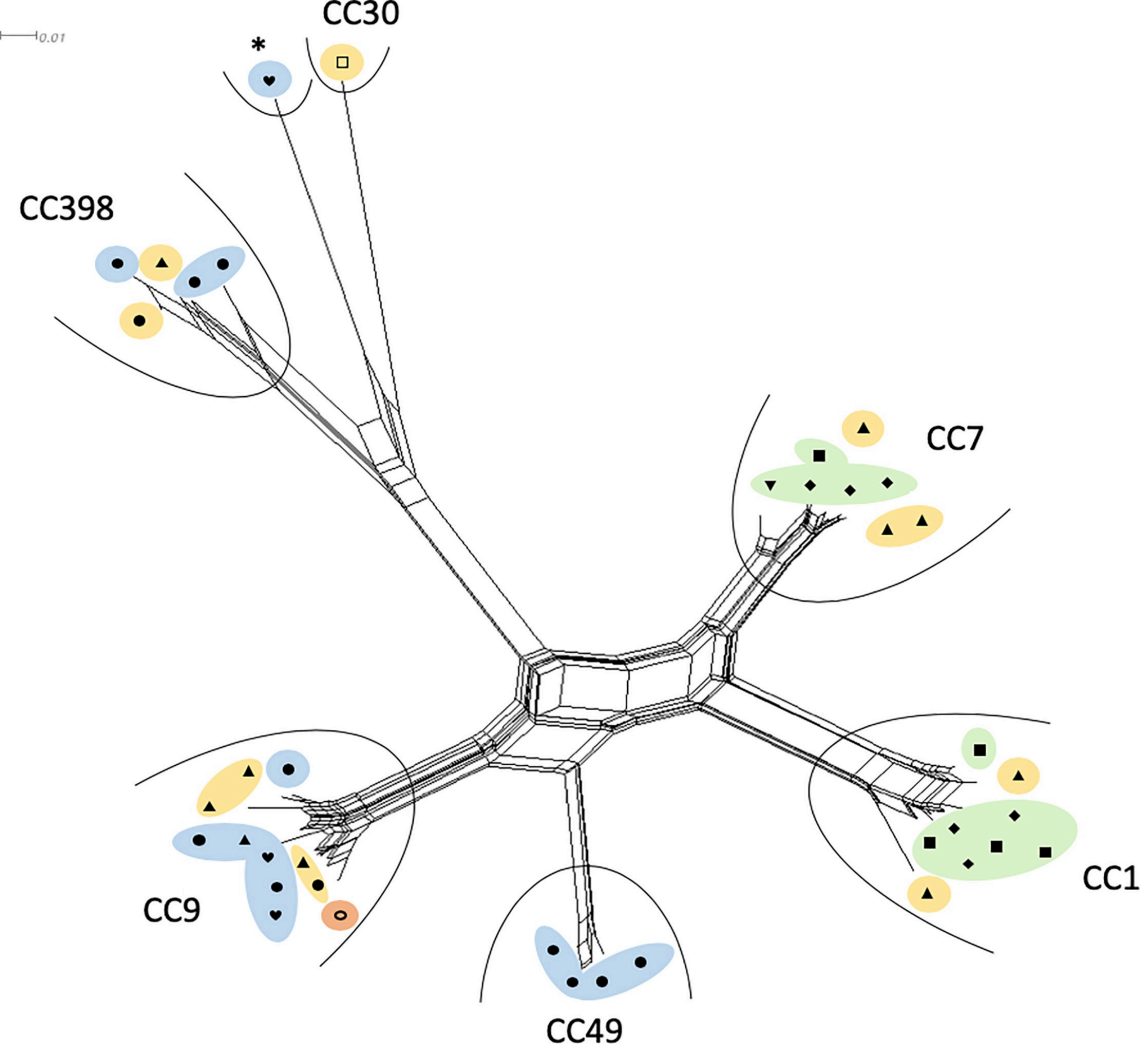
Splitstree showing the similarity of microarray hybridization profiles between the investigated *S*. *aureus* isolates. The corresponding clonal complexes are grouped by an arc. The source of isolation is labelled with following symbols: ear (▲), sternum (■), tongue (●), heart (♥), liver (□), rib bone (◆), forefoot (▼), intestine (◯). Each symbol represents a single isolate. The underlying colour indicates the source abattoir: Abattoir A (blue), Abattoir B (red), Abattoir C (yellow) and abattoir D (green). * No CC was assigned by the microarray. Isolates of *spa* type t015 assigned to CC45 have been previously reported [12,19,20].

Overall, 40 (8%) of the 524 sampled byproducts were positive (> 100 cfu/g) for *S*. *aureus*. Colonization of products ranged from 100 to 1800 CFU/g, with a mean of 360 CFU/g and a standard deviation of 479 CFU/g. Products, for which no *S*. *aureus* were detected, include hind foot, stomach, and bladder (Table 1). Parts with the highest prevalence were tongue (29%) and ear (24%), followed by rib bone (13%), sternum (9%), heart (7%) forefoot (2%) and liver (2%). The prevalence was lower than in other studies examining pork carcasses in Europe [15–17] or Asia [18]. *S*. *aureus* was found in only four of the six sampled abattoirs (Fig 1).

**Table 1:** Prevalence of *S*. *aureus* on sampled byproducts.

Organ	Positive samples^a^/total samples	Prevalence [%]
Sternum	5/56	9
Rib bone	7/56	13
Hind foot	0/56	0
Forefoot	1/56	2
Intestine	1/20	5
Heart	3/42	7
Liver	1/42	2
Stomach	0/56	0
Ear	10/42	24
Bladder	0/56	0
Tongue	12/42	29
**Total**	**40/524**	**8**

^a^>100 CFU/g

### Clonal complex and *spa* type

Of the 40 isolates obtained from pork byproducts, 39 could be assigned to a total of six clonal complexes (CC). Twelve *spa* types were associated with the samples (Table 2). The most frequent *spa* types were t091 (n = 9), t1491 (n = 8), t899 (n = 6) and t034 (n = 5). Other types that were represented to a lesser extent were t337 (n = 3), t208 (n = 2), t4049 (n = 2), t015 (n = 1), t1778 (n = 1), t1333 (n = 1), t2922 (n = 1) and t7439 (n = 1). The most prevalent CCs were CC9 (27.5%), CC1 (22.5%) and CC7 (22.5%). All other strains belonged to either CC398 (12.5%), CC49 (10%) or CC30 (2.5%), whereas one sample (t015 (2.5%)) could not be assigned to a CC by microarray analysis. Previous studies have assigned t015 strains to CC45 [12,19,20]. Johler et al. (2011) reported occurrence of CC9 (58.9%), CC49 (2.5%) and CC398 (28.2%) on pig carcasses in Switzerland. Similar prevalence was reported for porcine isolates from Denmark with CC398 (39%), CC30 (29%) and CC9 (27%) [21]. Strains belonging to CC9, CC30, CC49 and CC398 were found in slaughterhouses in Latvia [17] and in pigs and pig farmers in Switzerland [22]. CC1 has been associated with human infection [23] as well as pig farming [24]. An Italian study discovered overlapping of isolates found in abattoir workers and pork meat isolates, mainly for CC1 and CC398 [25]. CC7(t091) has regularly been linked to human infections [26,27] but appearance on pork meat has been observed as well [28–30] and the *spa* type has been linked with outbreaks in China [31]. In Europe CC1 (t1491) has been linked to human infections [32] or nasal colonization [33,34] and was found on pigs [21,28]. CC9 (t899) had already been identified in slaughtering pigs in Switzerland [35]. For CC45(t015), frequent occurrence as a human nasal colonizer has been reported [12,36], possibly linking this strain to processing contamination in the present study, as it was isolated from a heart and has only rarely been linked with pork [28]. An attribution of CCs to the respective source of isolation (body part) showed no difference between CCs present at outer body parts and those on inner organs (Fig 1). It could be hypothesized that inner organs were contaminated during meat processing. This is supported by the fact that not all CCs were found in all abattoirs. CC49 and CC45 were exclusively found in abattoir B, CC30 only in abattoir C. Other complexes appeared in two or more abattoirs, but none were present in all four abattoirs (Fig 1).

**Table 2:** Characteristics of 40 *S*. *aureus* isolates obtained from pork abattoir byproducts.

Clonal complex	*spa* type	Nr. of isolates	Resistance genes^a^	Enterotoxin genes^a^	Capsule type^a^	Comments ^a^
CC^b^	t015	1	*blaZ/I/R*	ND	8	*spa*-negative on microarray
CC1	t1491	8	*blaZ/I/R* (7)	*sea*^c^ (2), *seb* (3), *sec* (1), *seh* (3), *sel* (1), egc^d^ (2)	8	
	t1778	1	*qacC*	*sea*^d^	8	
CC7	t091	9	*aacA-aphD* (1)	*sea*^d^ (2), *seh* (1), egc (5)	8	
CC9	t337	3	*blaZ/I/R*, *fosB*	*seb* (1), *seh* (1), egc (1)	5	*fnbA*-negative (1)
	t899	6	*blaZ/I/R* (1), *fosB*	*seb* (2), *seh* (2), egc (2)	5	
	t2922	1	*blaZ/I/R*, *fosB*	egc	5	
	t7439	1	*blaZ/I/R*, *fosB*	ND	5	
CC30	t1333	1	*fosB*	*seb*, *seh*	8	
CC49	t208	2	*blaZ/I/R*	*sea*^d^ (1)	5	
	t4049	2	*blaZ/I/R*	*sea*^d^ (1), egc (1)	5	
CC398	t034	5	*blaZ/I/R* (2), *vgaA* (2), *tetK* (1), *tetM*	*sea*^d^ (2), *seb* (1), *seh* (1), egc (1)	5	

^a^ If not all isolates assigned to the respective *spa* type harbored a gene, the number of positive isolates is indicated in brackets.

^b^ No CC was assigned by the microarray. Isolates of *spa* type t015 assigned to CC45 have been previously reported [12,19,20].

^c^ allelic variant *sea* N315

^d^ Enterotoxin gene cluster (*egc*) containing genes: *seg*, *sei*, *sem*, *sen*, *seo*, *seu*

One of the isolates (t015) appeared as *spa*-negative on the DNA microarray, while another (t337) was *fnbA*-negative in the microarray. This may hamper recognition by rapid *S*. *aureus* identification tests relying on latex agglutination via binding with human IgG or fibrinogen, respectively (e.g. Staphaurex by Remel, Oxoid AG, Pratteln, Switzerland) [37]

### Resistance genes

Among the tested antibiotic resistance genes, *blaZ/I/R*, *qacC*, *fosB*, *vgaA*, *tetK/M*, and *aacA-aphD* were found. As depicted in Table 2, CC398 appeared to exhibit the most heterogeneous resistance profile compared to other complexes. For CC398 isolates, resistance genes *blaZ/I/R* conferring *β*-lactam resistance were detected as well as *vgaA* contributing to streptogramin-A resistance and the tetracycline resistance markers *tetK* and *tetM*.

No MRSA were detected among the *S*. *aureus* isolates investigated in this study. Occurrence of MRSA has been scarcely described in CC7 or CC49 [38]. In contrast, MRSA have been found regularly in CC1, CC9, CC30, CC45 and CC398 [38,39]. CC398 has lately received a lot of attention as a source of livestock-associated MRSA in the Netherlands, Germany, Belgium, Italy, Austria, Spain, the USA and Australia [40–43]. The presence of tetracycline resistance genes *tetK* and *tetM* in CC398 characterized in our study reinforce the potential of these strains to act as precursors for CC398 LA-MRSA emergence, as CC398 MRSA are always tetracycline resistant [44].

Other resistance genes appearing in various strains were the *fosB* gene coding for a metallothiol transferase, *qacC* the quaternary ammonium compound resistance protein C and *aacA-aphD* conferring gentamicin and tobramycin resistance.

### Enterotoxin genes

The studied set of isolates displayed a variety of enterotoxin genes, which were heterogeneously distributed within clonal complexes and *spa* types. As illustrated in Table 2, *sea* (N315) was present in CC1, CC7, CC49, and CC398. The gene coding for enterotoxin B (*seb*) was found in CC1, CC9, CC30, and CC398. The *seh* gene was distributed across CC1, CC7, CC9, CC30, and CC398. The most prevalent toxin genes were the *egc* encoded genes *seg*, *sei*, *sem*, *sen*, *seo* and *seu*, which were detected in 11 strains belonging to CC1, CC7, CC9, CC49, and CC398. On conventional cuts of pork meat similar enterotoxin profiles have been reported [10,45]. Interestingly, only one strain isolated from a heart harbored the *sec* and *sel* genes. Only two strains (*spa* types t015 & t7439) did not harbor any of the tested enterotoxin genes.

### Similarity of genomic profiles

Visualization of similarity of microarray hybridization profiles in a Splitstree (Fig 1) revealed an association of certain CCs and abattoirs. No association of CCs with particular body parts or outer/inner organs was observed. However, *S*. *aureus* from certain body parts were associated with certain abattoirs, e. g. *S*. *aureus* were only detected in sternum and rib bone samples originating from abattoir D. It could be hypothesized that such isolates stem from post mortem contamination during the slaughtering and meat handling process, rather than from the animal source.

## Conclusion and outlook

Sampling of pork byproducts in Switzerland demonstrated low prevalence of *S*. *aureus*. Microarray based genetic profiling of 40 *S*. *aureus* isolates revealed a diverse population structure. No MRSA were detected. A variety of enterotoxin genes was found distributed over almost all clonal complexes. Overall, the isolates did not differ considerably from those found in previous studies in conventional pork meat cuts.

Our findings suggest that occurrence of *S*. *aureus* on byproducts was linked to contamination during the slaughtering process in some abattoirs. Adequate handling of these processing sidestreams should ensure proper quality and therefore minimize product loss.

## Supporting information

S1 Table Microarray profile raw data. The outcome of every hybridization reaction is shown as either positive, negative, or ambiguous as analyzed by the ArrayMate reader (Alere).
(XLSX)
